# Role of B lymphoma Mo-MLV insertion region 1 in the oncogenic behavior of retinoblastomas

**Published:** 2013-03-15

**Authors:** Ruojin Ren, Weiwei Liu, Li Huang, David Tai Li Liu, Kwong Wai Choy, Jitong Shi, Junyang Zhao, Bowen Zhao, Ming Guan, Carol L. Shields, Chi Pui Pang, Bin Li, Gary Hin Fai Yam

**Affiliations:** 1 Department of Ophthalmology & Visual Sciences, The Chinese University of Hong Kong, Hong Kong; 2 Obstetrics & Gynaecology, The Chinese University of Hong Kong, Hong Kong; 3 Beijing Institute of Ophthalmology, Beijing TongRen Hospital Eye Center, Capital Medical University, Beijing, China; 4 Department of Laboratory Medicine, Huashan Hospital, Fudan University, Shanghai, P.R. China; 5 Ocular Ocology Service, Wills Eye Institute, Thomas Jefferson University, PA; 6 Tissue Engineering and Stem Cell Research Group, Singapore Eye Research Institute, 11 Third Hospital Avenue, Singapore, Singapore

## Abstract

**Purpose:**

This study investigated the relationship between B lymphoma Mo-MLV insertion region 1 (BMI-1)—a polycomb protein for stem cell self-renewal and proliferation—and the clinicopathological parameters of human retinoblastomas, including differentiation status and retinal tissue invasion, as well as the effects of BMI-1 on retinoblastoma Y79 cells.

**Methods:**

Thirty-four archived human retinoblastoma samples were recruited for BMI-1 immunohistochemistry. The percentage of BMI-1-expressing cells was scored by independent pathologists and the data were correlated with the clinical features. Y79 cells were transfected to overexpress or specifically inhibit BMI-1 for cell proliferation, propidium iodide cell cycle and terminal deoxynucleotidyl transferase dUTP nick end labeling (TUNEL) apoptosis analyses, multicellular sphere formation assay, and gene expression study.

**Results:**

BMI-1 was widely expressed in human retinoblastomas. Higher percentages of BMI-1-expressing cells were selectively limited to undifferentiated tumors and those tumors undergoing invasion to the optic nerve and choroid. However, there was no difference in BMI-1 expression in retinoblastoma retinas with or without tumor invasion. In Y79 cells, BMI-1 stimulated cell proliferation and suppressed apoptosis with reduced p14ARF and p16INK4 expression, along with upregulation of proliferating cell nuclear antigens cyclin D1 and D2. In contrast, silencing BMI-1 reversed these changes. It also upregulated CHX10 and Rx, but not other retinal development-related genes, including nestin and neurofilament M.

**Conclusions:**

Our work indicates that BMI-1 might render important oncogenic property of retinoblastomas and it could be a therapeutic target for the cancer treatment.

## Introduction

Retinoblastoma (OMIM 180200) is the most commonly encountered pediatric intraocular tumor (3% of childhood cancer) and affects about 1 in 15,000 live births worldwide [1,2]. It is highly malignant and mostly manifested in the first five years of life and causes death in 50% of affected children worldwide. The mortality, however, varies from less than 5% of children in the United States and other developed countries with advanced medical care to more than 70% in some developing countries [3]. More than 50% of retinoblastoma cases are sporadic and mainly caused by *RB1* gene mutation [4-7]. Despite intensive pathological, genetic, and epigenetic studies, the histogenesis of retinoblastoma is not well defined [8-11]. It is debatable whether retinoblastoma is generated from naturally death-resistant retinal precursor cells or *RB1*-deficient retinoblasts undergoing p53 pathway inactivation-mediated apoptosis and exit of the cell cycle [12,13]. Expression of retinal development–related genes, including *NESTIN*, *Rx*, *PAX6*, and *CHX10*, shows that retinoblastoma can be derived from undifferentiated retinoblasts [14]. A subpopulation of retinoblastoma cells also displays immunoreactive ATP-binding cassette transporter glycoprotein G2, aldehyde dehydrogenase I, stem cell antigen-1, p63, and B lymphoma Mo-MLV insertion region 1 (BMI-1), indicating the properties of self-renewal and proliferation capacity of cancer stem cells [15]. Moreover, BrdU retention and the expression of OCT3/4, NANOG, and Musashi-1 have been detected in retinoblastoma tumors and cell lines, further suggesting that they contain cells with embryonic stem-like properties [16].

BMI-1 belongs to the polycomb group proteins, which form a multiprotein complex and are involved in transcriptional regulation [17,18]. It is an oncogene that regulates cell proliferation and senescence through the repression of the Ckdn2a locus, which encodes the p16Ink4a and p14Arf tumor suppressor proteins [19,20]. It is implicated in the self-renewal and proliferation of various types of somatic stem cells, including neural, hematopoietic, and prostate cells [21-23]. Deregulation of BMI-1, such as in gene amplification and protein overexpression, has been linked to the tumorigenesis of leukemia and solid tumors like oligodendroglial tumors and prostate, breast, and colorectal cancers [20,23-27]. In some instances, BMI-1 overexpression has been attributed to unfavorable prognosis in patients with squamous cell carcinoma of the tongue, hepatocellular carcinoma, and prostate and pancreatic cancers [28-31]. In nasopharyngeal carcinoma, the five-year survival rate of patients was over 80% if BMI-1 was negative, but only 47% if it was positive [32]. In this study, we investigated BMI-1 expression and the clinicopathological parameters of human retinoblastomas. The effect of BMI-1 overexpression and reduction on the growth, cell cycle changes, and apoptosis of Y79 cells was examined.

## Methods

### Retinoblastoma cases

The study protocol of human retinoblastoma archive specimens was approved by the Ethics Committee for Human Research, Beijing Tongren Hospital, Capital Medical University, China and adhered to the tenets of Declaration of Helsinki. Paraffin-embedded retinoblastoma eyeballs from 34 patients were archived in Beijing Tongren Hospital from 2007 to 2008. These patients had no associated medical and family history. All specimens were serially sectioned along the pupillary–optic nerve head axis and the sections were used for immunohistochemistry.

### Immunohistochemistry of BMI-1 on archived sections

Immunoreactive BMI-1 was detected using the labeled horseradish peroxidase method. On paraffin sections, antigen was retrieved by trypsin and endogenous peroxidase inactivated by 0.3% hydrogen peroxide in methanol. After blocking with 5% normal goat serum and 0.1% Triton X-100, the section was incubated with or without 0.5 to 1 μg/ml antihuman BMI-1 monoclonal antibody (Clone F6, Millipore, Billerica, MA). Control sections were incubated with buffer without primary antibody. Following PBS rinses, sections were treated with goat anit-mouse immunoglobulin horseradish peroxidase conjugate (Jackson ImmunoRes Lab, West Grove, PA). Signals were revealed by 3,3'-diaminobenzidine (DAB) reduction and examined under light microscopy (DMRB, Leica, Wetzlar, Germany) equipped with Spot RT color system (Diagnostic Instruments Inc., Sterling Heights, MI). Stained sections were examined by two masked pathologists (RRJ, LTL). In 10 random images taken at 50× magnification, the percentage of BMI-1 positive cells in tumor or retinal layers was graded as (0) no staining, (1) <5%, (2) 5%–25%, (3) 25%–50%, or (4) >50%.

### Expression constructs of BMI-1

BMI-1 expression construct was prepared by cloning a 990 bp *EcoR1*/*Xho1* fragment encompassing full-length 981 bp open reading frame of wild-type human *BMI-1* to *EcoR1/Xho1* site of a mammalian expression vector pCMV-HA (Clontech, Mountain View, CA) to create pHA-BMI-1. Alternatively, for specific knockdown, synthesized 64 bp oligonucleotide containing human BMI-1 small interfering RNA (siRNA) sequence (5′-ATG AAG AGA AGA AGG GAT T-3′, position 269–287 bp from the start codon) was cloned into the HindIII/BglII site in the pSuper vector (Oligoengine, Seattle, WA) to generate pSuper-BMI-1. All constructs were verified by direct sequencing.

### Cell transfection

Y79 cells (American Tissue Cell Collection, Manassas, VA) were maintained in RPMI-1640 (Invitrogen, Carlsbad, CA) supplemented with 10% fetal bovine serum (FBS, Invitrogen), 100 U/ml penicillin G, and 100 μg/ml streptomycin sulfate at 37 °C under humidified conditions in 5% CO_2_ balanced with air. The BMI-1 construct was transfected to cells at 5×10^5^ cells/ml by Lipofectamine 2000 (Invitrogen) at a ratio of 3 µl reagent per µg DNA in Opti-MEM® Reduced Serum Medium, GlutaMAX™ (Invitrogen). One day after transfection, the cells were maintained in 80 µg/ml Geneticin-418 (Invitrogen) for 10 days. Drug-resistant cells were pooled for protein and RNA analyses.

### Cell growth, viability, and apoptosis assays

Transfected cells at a density of 5×10^5^ cells/µl were cultured in a six-well plate. Every 24 h, 200 µl of cell suspension was collected for for tetrazolium dye (MTT) cell viability/proliferation assay. Terminal deoxynucleotidyl transferase dUTP nick end labeling (TUNEL) assay using ApopTaqIn Situ Apoptosis Detection Kit (Oncor, Gaithersburg, MD) was performed on paraformaldehyde-fixed cytospinned cells. The TUNEL-positive and 4',6-diamidino-2-phenylindole (DAPI)-labeled cells were counted in 10 random images captured under fluorescence microscopy with a 20x objective. The apoptosis rate was determined as the percentage of TUNEL-positive cells. All experiments were carried out in triplicate.

### Multicellular sphere assay

Single transfected cells at 50 cells/ml were passed through 40 µm nylon mesh and incubated in a culture dish (100 mm diameter) in serum-free RPMI-1640 medium supplemented with 10 ng/ml basic fibroblast growth factor (Invitrogen). After 7 days, the culture was examined for sphere formation and the number and sizes of spheres were recorded. Cell proliferation was assayed by BrdU incorporation for 2 h, followed by immunofluorescence using anti-BrdU antibody (Abcam, Cambridge, UK), followed by Alexa 488-conjugated immuneglopbulin G (IgG) secondary antibody (Jackson ImmunoRes Lab, West Grove, PA).

### Propidium iodide cell cycle analysis

Transfected cells were harvested and fixed with 70% ice-cold ethanol with vigorous shaking to prevent cell clustering. The cells were incubated with 50 μg/ml propidium iodide (Invitrogen) in the presence of 0.1 mg/ml RNase A (Sigma) and 0.05% Triton X-100. After washes, the cells were resuspended in PBS for flow analysis using a FACS Calibur Flow Cytometer (BD).

### Western blotting

The cells were lysed at 2.5×10^6^ cells/µl in radioimmunoprecipitation buffer containing 50 mM Tris-HCl (Sigma, St Louis, MI), 150 mM sodium chloride, 1% Nonidet P40, 0.25% sodium deoxycholate, protease inhibitor cocktail (Roche, Basel, Switzerland), and 1 mM phenylmethyl sulfonylfluoride. The clear supernatant was denatured in 2% sodium dodecyl sulfate (BioRad, Hercules, CA) and 50 mM DL-dithiothreitol. Proteins (equivalent to 2×10^5^ cells) were resolved by 10% or 13% sodium dodecyl sulfate–polyacrylamide gel electrophoresis and analyzed by enhanced chemiluminescence using antibodies against HA (Millipore), BMI-1 (Millipore), p14ARF (Santa Cruz Biotechnol., Santa Cruz, CA), p16INK4a (Santa Cruz), cyclin D1 (Santa Cruz), cyclin D2 (Santa Cruz), proliferating cell nuclear antigen (PCNA, Sigma), caspase-3 (Millipore), and β-actin (Sigma).

### Expression of ribonucleic acid

Total RNA was extracted with RNeasy kit (Qiagen, Valencia, CA), quantified and reverse transcribed with random primer (Roche) and SuperScript™ III reverse transcriptase (Invitrogen). Semiquantitative PCR was performed with specific primers for *CHX10*, *Rx*, *NESTIN*, *neurofilament-M* and *glyceraldehyde phosphate dehydrogenase* (Table 1). PCR products were resolved by 2% agarose gel electrophoresis and analysed by Quantity One Image Analysis. Gene expression was normalized with glyceraldehyde phosphate dehydrogenase. Three independent experiments were performed.

**Table 1:** Specific primer sequences for gene expression study.

**Gene**	**GenBank Accession No.**	**Primer sequence (5’-3’)**
*CHX10*	NM_182894	F: GAGAAGGCATTCAACGAAGC
		R: CATACTCCGCCATGACACTG
*Rx*	NM_013435	F: AGCGAAACTGTCAGAGGA
		R: TCATGCAGCTGGTACGTGGTGA
*NESTIN*	NM_006617	F: CCTGGGAAAGGGAGAGTACC
		R: GGATGAGGCAGAGCTGAATC
*neurofilament-M*	NM_005382	F: ATCGGTAAAGGTGCACTTG
		R: TCTACCTCCCCATTGACAGC
*glyceraldehyde phosphate dehydrogenase*	BC_014085	F: GAAGGTGAAGGTCGGAGT
		R: GAAGATGGTGATGGGATTTC

### Statistical analyses

The analysis was performed using a commercially available statistical software package (SPSS for Windows, version 17.0, SPSS, Chicago, IL). The data were presented as mean and standard deviation, as well as in median and ranges. For the comparison of study groups, the Mann–Whitney U test was applied for unpaired samples. If there was one variable possibly associated with the dependent parameter, a Spearman correlation analysis was performed. A value of p<0.05 was considered as statistical significance.

## Results

### Demography and retinoblastoma pathology

A total of 34 retinoblastoma specimens were recruited in this study. They were collected at Beijing Tongren Hospital from April 2007 to April 2008 (Table 2). The patients had no associated medical or family history. Twenty-one were males and 15 were females, and the average age was 2.27±1.13 years. There were 31 unilateral retinoblastomas (15 left and 16 right eyes) and 5 bilateral retinoblastomas. Leukocoria was commonly presented (31 eyes), while others exhibited pink eye (2 eyes), esotropia (1 eye), reduced vision with esotropia (1 eye), and leukocoria with esotropia (1 eye).

**Table 2:** Demographic and clinical characteristics of studied retinoblastoma cases.

Gender	Male 58.3%; female 41.7%
Laterality	Unilateral: 31/34 (91.2%)
	right eye: 16/31; left eye: 15/31
	Bilateral: 5/34 (14.7%)
Mode of presentation	Leukocoria: 31/34 (91.2%)
	Pink eye: 2/34 (5.9%)
	Esotropia: 1/34 (2.8%)
	Reduced vision with esotropia: 1/34 (2.9%)
	Leukocoria with esotropia: 1/34 (2.9%)
Extraocular invasion	Optic nerve invasion: 22/34 (64.7%)
	Choroidal invasion: 11/34 (32.3%)
	focal: 7/11
	massive: 4/11
	No optic nerve or choroidal invasion: 11/34 (32.3%)
	Both types of invasion: 8/34 (23.5%)
RB differentiation	Undifferentiated: 24/34 (70.6%)
	Differentiated: 10/34 (29.4%)

### BMI-1 expression and retinoblastoma differentiation

**Figure 1:**
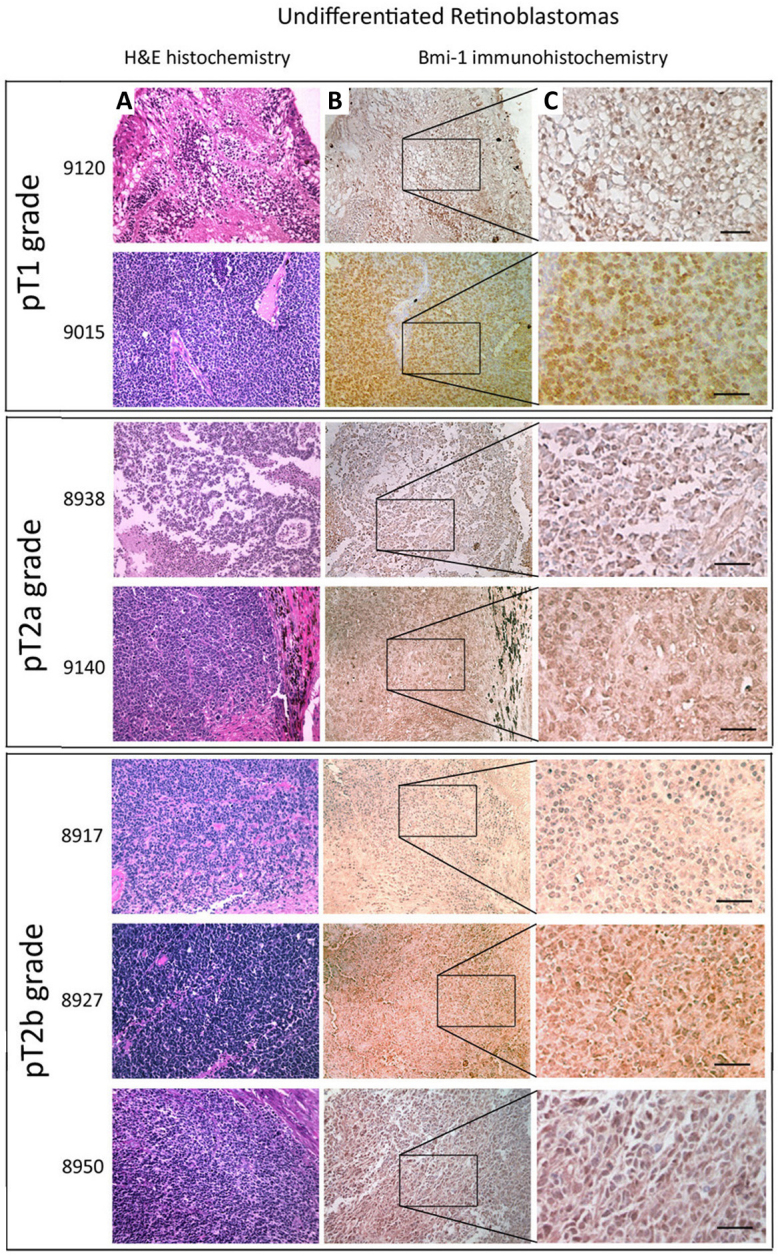
Representative light microscopy pictures of staining of B lymphoma Mo-MLV insertion region 1 (BMI-1) antigen in undifferentiated retinoblastomas grouped according to Tumor, Nodes, Metastasis (TNM) classification. **A**: Retinoblastoma sections were stained by hematoxylin and eosin.**B:** Magnified images in **C**: intense nuclear BMI-1 expression in undifferentiated retinoblastomas. Scale bars: 50 µm.

**Figure 2:**
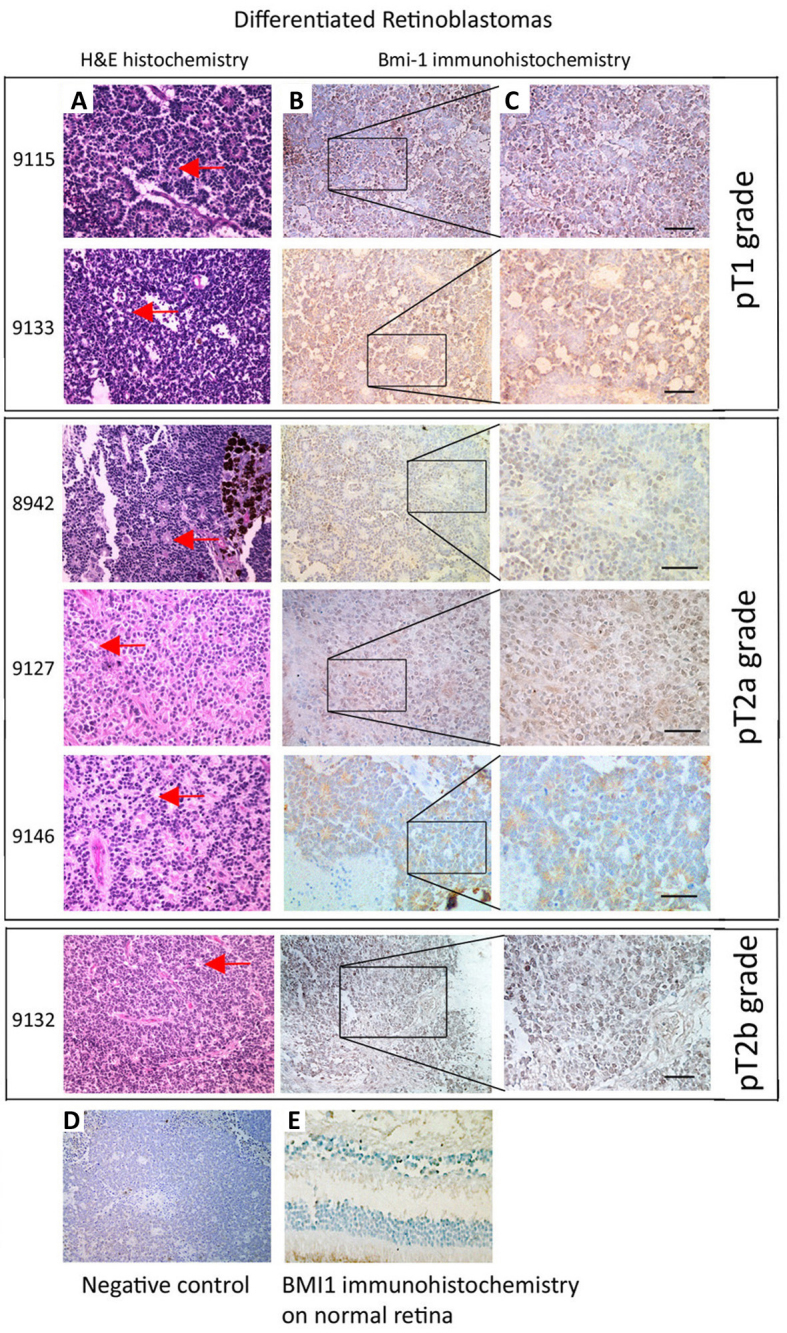
Representative light microscopy pictures of staining of B lymphoma Mo-MLV insertion region 1 (BMI-1) antigen in differentiated retinoblastomas grouped according to Tumor, Nodes, Metastasis (TNM) classification. **A**: Hematoxylin and eosin staining. Closed red arrows in A show the Flexner-Wintersteiner rosettes. **B:** Magnified images in **C**: weak to moderate BMI-1 expression in differentiated retinoblastomas. **D**: Background staining of BMI-1 in retinoblastoma 8927 with incubation of primary antibody. **E**: Negligible BMI-1 expression in a normal human retina section. Scale bars: 50 µm.

Twenty-four tumors without Flexner-Wintersteiner rosettes and showing small-sized cells with a large nucleus-to-cytoplasm ratio were classified as undifferentiated type (column A in Figure 1). Ten tumors were characterized as differentiated with typical Flexner-Wintersteiner rosettes with cells surrounding the central lumen (column A in Figure 2). Homer-Wright rosettes bearing eosinophilic neurofibrillary cores were occasionally observed. Intratumor necrosis was infrequently observed.

Nuclear BMI-1 staining was present in the majority of retinoblastoma specimens (33/34 tumors, 97.1%). The staining intensity varied with the differentiation status. Distinct BMI-1 expression was observed in >50% tumor cells in 13 undifferentiated retinoblastoma cases (13/24, 54.2%; representative pictures in column B of Figure 1, magnified images in column C), but not in any differentiated cases (0/10, 0%; Table 3). Ten undifferentiated cases (10/24, 41.7%) had milder BMI-1 staining (<50% tumor cells). Only one case was BMI-1 negative. On the other hand, BMI-1 was not clearly observed in the differentiated tumors. All cases exhibited BMI-1 immunoreactivity in <25% tumor cells (representative pictures in column B of Figure 2, magnified images in column C). Hence, the expression of BMI-1 was shown to be significantly associated with undifferentiated retinoblastomas (p=0.05, Mann–Whitney U test).

**Table 3:** BMI-1 expression and clinicopathological features of retinoblastomas.

	Grading of % BMI-1 positive cells				
	*0	1	2	3	4
**(A) Rb differentiation**					
1. Undifferentiated (n=24)	1	5	5	7	6
2. Differentiated (n=10)	0	3	7	0	0
p=0.05 (Mann–Whitney U test)					
**(B) Rb retinas**					
1. Undifferentiated Rb (n=17)	3	6	8	0	0
2. Differentiated Rb (n=10)	1	5	4	0	0
p=0.86 (Mann–Whitney U test)					
**(B1) Outer nuclear layer**					
1. Undifferentiated Rb (n=17)	3	6	8	0	0
2. Differentiated Rb (n=10)	1	6	3	0	0
p=0.49 (Mann–Whitney U test)					
** (B2) Inner nuclear layer**					
1. Undifferentiated Rb (n=17)	3	4	7	3	0
2. Differentiated Rb (n=10)	1	2	5	1	0
p=0.24 (Mann–Whitney U test)					
**(C) Tumor invasion**					
1. No invasion (n=9)	2	4	2	1	0
2. Invasion (n=25)	1	3	9	6	6
(i) to optic nerve only (n=14)	0	2	7	3	2
(ii) to choroid only (n=3)	1	1	1	0	0
(iii) to both (n=8)	0	0	1	3	4
**(D) Optic nerve invasion (n=22)**					
1. To optic nerve head (n=13)	0	2	7	4	0
2. Past lamina cribrosa (n=9)	0	0	1	3	6

p=0.37 (Mann–Whitney U test)* 0 represents no staining; 1: <5%; 2: 5%–25%; 3: 25%–50%; 4: >25% BMI-1 stained cells

**Figure 3:**
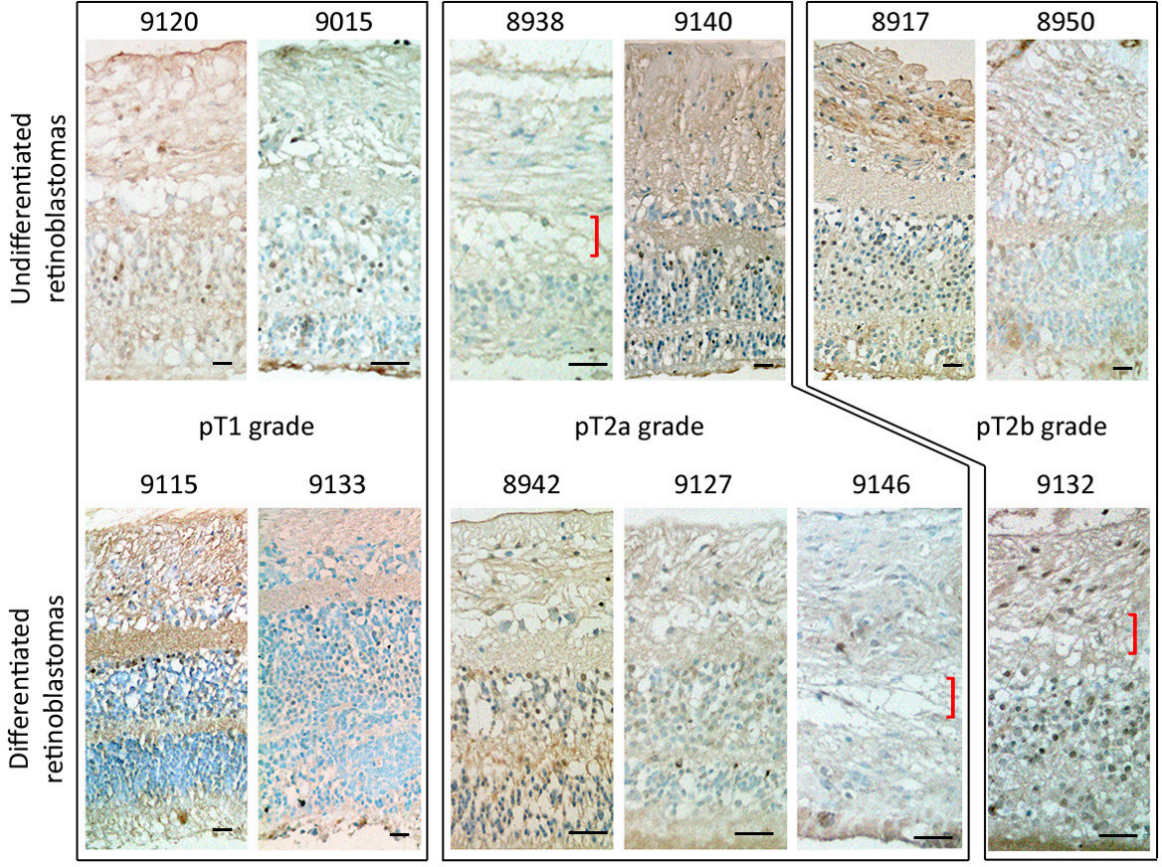
Representative light microscopy pictures of staining of B lymphoma Mo-MLV insertion region 1 (BMI-1) antigen in human retinoblastoma retinae. (Upper panel) Undifferentiated retinoblastomas and (lower panel) differentiated retinoblastomas grouped according to TNM classification. Some specimens had an indistinct inner nuclear layer (marked by red brackets). There was no consistent difference of BMI-1 expression in retinas of undifferentiated and differentiated retinoblastomas. Scale bars: 50 μm.

We studied BMI-1 expression in 27 retinal tissues adjacent to retinoblastoma (Table 3). Notably, it was not widely detected in all retinas. Most retinal tissue next to undifferentiated retinoblastomas had positive BMI-1 staining in <25% retinal cells (14/17, 82.3%; representative pictures in upper panel of Figure 3). A similar observation was made for differentiated cases (9/10, 90%; representative pictures in lower panel of Figure 3). The remaining was BMI-1 negative. Hence, there was no significant correlation between BMI-1 expression in retina in terms of the differentiation status of retinoblastomas (p=0.86, Mann–Whitney U test). Furthermore, altered retinal layers, including an indistinct inner nuclear layer, was infrequently observed in both differentiated and undifferentiated tumors (marked by red brackets in Figure 3). There was no association between BMI-1 expression in the outer and inner nuclear layer with retinoblastoma differentiation status (p=0.49 and p=0.24, respectively; Table 3).

### BMI-1 expression and retinoblastoma invasion to posterior tissues

In our 34 studied cases, 9 retinoblastomas remained in situ and no invasion to adjacent retinal tissue was observed (9/34, 26.5%; pT1 grade in Pathologic Classification of TNM [Tumor, Node, Metastasis] Classification for Retinoblastoma, Table 3). For the remaining 25 cases showing tumor cell invasion, involvement of the optic nerve was only detected in 14 retinoblastomas (38.9%; pT2a grade), 3 were choroid only (8.3%), and 8 were invaded to both optic nerve and choroid (22.2%; pT2b grade). The percentage of BMI-1 positive cells was significantly greater in retinoblastomas with invasion than those without (p=0.0023, Mann–Whitney U test; Figure 1 and Figure 2; Table 3). Among these malignant cases, more BMI-1 expressing cells were found in tumors invading both the optic nerve and choroid (p=0.0005), whereas fewer were found in tumors with invasion to either location. For retinoblastomas showing optic nerve invasion (n=22), 13 were in the optic nerve head (pT2 grade) and 9 were extended to the posterior surface of the lamina cribrosa (pT3 grade) and percentage of BMI-1 positive cells was similar in both categories (Table 3).

### Effect of BMI-1 on retinoblastoma cell growth and proliferation

**Figure 4:**
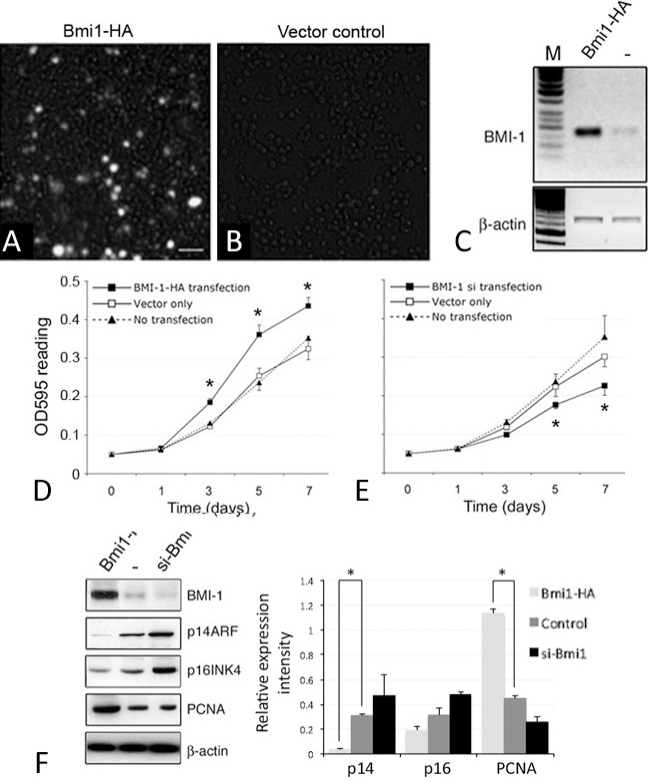
B lymphoma Mo-MLV insertion region 1 (BMI-1) expression altered retinoblastoma 480 Y79 cell growth. **A**-**C**: Immunostaining and reverse transcription (RT)–PCR analyses show marked increase of BMI-1 transcript and protein in Y79 cells after transfection with pCMV-HA/BMI-1 compared with vector control. **D** and **E**: MTT assay showing Y79 cell viability and proliferation was promoted by BMI-1 overexpression and reduced by siRNA-mediated inhibition of *Bmi-1*. **F**: Gene expression analysis of p14, p16, and PCNA in pCMV-HA/BMI-1, pSuper-BMI-1-si, and vector-transfected Y79 cells. *p<0.05, paired Student *t* test. Scale bar: 25 μm.

Human retinoblastoma Y79 cells were transfected with pCMV-HA/BMI-1 for BMI-1 overexpression or by pSuper-BMI-1-si for specific BMI-1 suppression. Stable transfectants were obtained after G418 selection for 10 days (Figure 4A). Cells transfected with empty vectors (pCMV-HA or pSuper) and nontransfected cells served as controls (Figure 4B).

**Figure 5:**
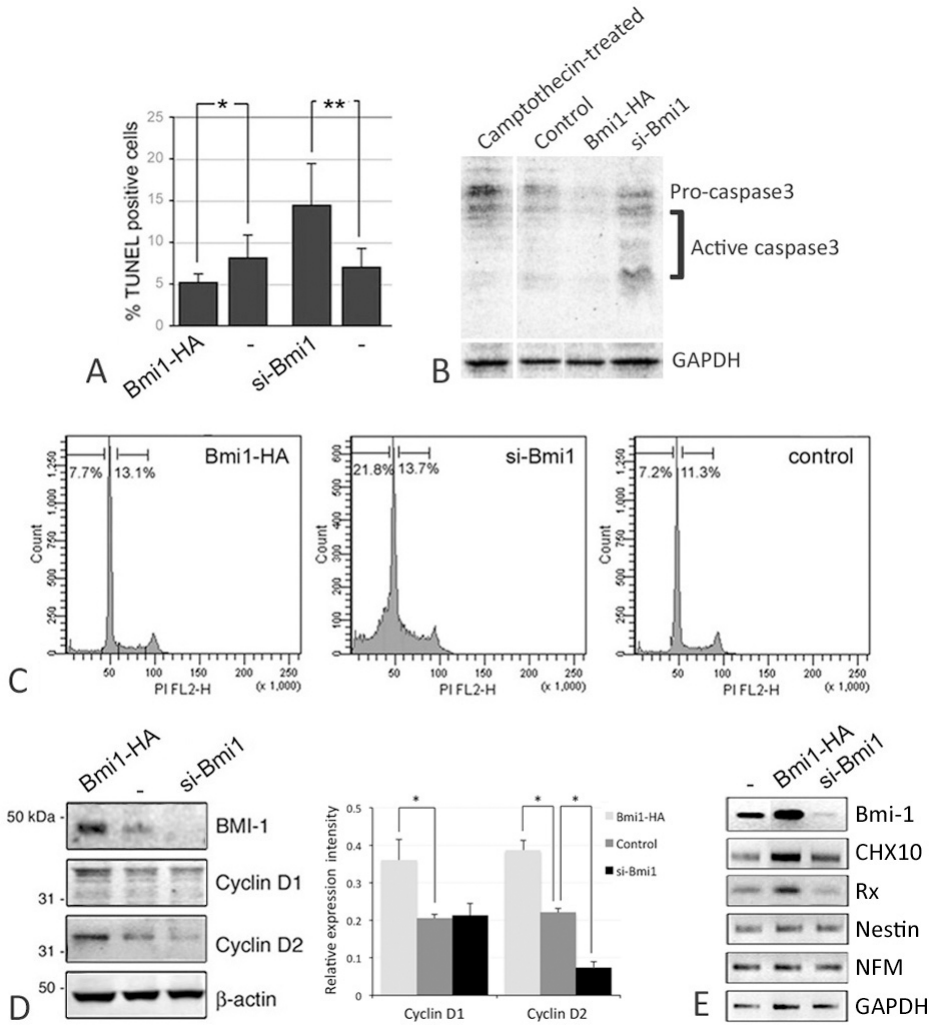
Influence of B lymphoma Mo-MLV insertion region 1 (BMI-1) on apoptosis and gene expression of Y79 cells. **A**: TUNEL assay showing increase of apoptosis rate after siBmi1 transfection to Y79 cells. A mild reduction of apoptosis was seen with BMI-1-HA expression (n=3, mean±standard deviation). *p<0.01; **p<0.005. **B**: Western blotting of pro- and active caspase-3 in Y79 cell lysate after transfection. Cells treated with camptothecin (10 μM) for 12 h served as the positive control of apoptosis. **C**: Propidium iodide cell cycle analysis of transfected Y79 cells by flow cytometry. **D**: Western blotting and band densitometry analysis of BMI-1, cyclin D1 and D2 in Y79 cells after transfection. *p<0.05, paired Student *t* test. **E**: Expression of CHX10, Rx, nestin, and *neurofilament-M* expression in transfected Y79 cells.

Increased BMI-1 transcript and protein levels in Y79 cells with pCMV-HA/BMI-1 transfection were confirmed by semiquantitative PCR and western blotting of the HA epitope (Figure 4C,F). Nontransfected cells exhibited negligible HA staining. BMI-1-HA transfected Y79 cells showed increased cell viability and a shorter doubling time (17.9 h) when compared to nontransfected or mock-transfected control cells (both were 22.3 h; Figure 4D). They formed more multicellular spheres in 7 days than pSuper-BMI-1-si and nontransfected cells. The sphere size was generally greater and there were more spheres with a diameter greater than 100 µm in BMI-1-HA-transfected Y79 cells (63%) than nontransfected cells (40%). The BMI-1-transfected cells exhibited higher expression of PCNA (Figure 4F) and BrdU incorporation. The apoptosis rate, determined by the percentage of TUNEL-positive cells, was significantly decreased with BMI-1-HA expression (5.2±1%) when compared to control (8.2±2.7%; p=0.023, paired Student *t* test; Figure 5A). This observation was substantiated in the reduced expression of pro- and active caspase-3 in western blotting (Figure 5B). Using propidium iodide cell cycle analysis, there was no significant difference of S-phase population in BMI-1-HA-expressing Y79 cells (13.1%) compared to mock-transfected control cells (11.3%; Figure 5C).

**Figure 6:**
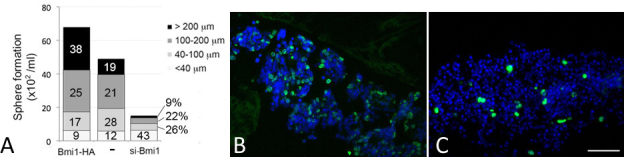
Effect of B lymphoma Mo-MLV insertion 486 region 1 (BMI-1) on multicellular sphere formation of Y79 cells. **A**: Single transfected cells were cultured for 7 days to form multicellular spheres. The number and size of spheres were quantified. In triplicated experiments, the mean percentage of spheres having with diameters was indicated. **B** and **C**: Cell proliferation assay by BrdU incorporation for Y79 cells transfected with BMI-1-HA. **B**: BMI-1-HA and **C**: siBmi 1. Scale bar: 100 μm.

In contrast, siRNA-mediated inhibition of BMI-1 impaired Y79 cell growth (Figure 4E). The cells showed longer cell doubling time (29 h) when compared to nontransfected or mock-transfected control cells (both were about 23.1 h). Further, they exhibited less multicellular sphere formation (less than one-third that of nontransfected cells; Figure 6A). A set of spheres (43%) were smaller in size (<40 µm in diameter). BrdU incorporation was also reduced (Figure 6C). There were more TUNEL-positive cells, and the apoptosis rate (14.5±5%) was significantly higher than in mock-transfected control cells (7±2.3%; p=0.0017, paired Student *t* test; Figure 5A). This was accompanied by an increased expression of active caspase-3 (Figure 5B). Propidium iodide cell cycle analysis showed that BMI-1 repression increased apoptotic cells (21.8%) when compared with mock-transfection (7.2%). There was no effect on S-phase alteration (13.7% in si-BMI-1 cells versus 11.3% in control; Figure 5C).

Western blotting showed that BMI-1 induced cyclin D1 (~37 kDa) and D2 (~34 kDa) expression (Figure 5D) and reduced cyclin-dependent kinase (CDK) inhibitor p14ARF and p16INK4 expression (Figure 4F). In contrast, its suppression by specific RNA interference caused a reduction in cyclin D1 and cyclin D2 (Figure 5D), accompanied by induction of p14ARF and p16INK4 (Figure 4F). BMI-1 also affected the expression of retinal development markers. The expression of *CHX10* and *Rx* was elevated with its ectopic expression in Y79 cells and reduced after the specific knockdown (Figure 5E). However, it did not affect the expression of *Nestin* and *neurofilament M*.

## Discussion

Increased expression of BMI-1 has been implicated in cancers, including colorectal, non-small cell pulmonary, hepatocellular, prostate, and breast cancer, medulloblastoma, multiple myeloma, and neuroblastoma [33-36]. In this study, we detected BMI-1 upregulation in undifferentiated human retinoblastomas when compared to differentiated cases (p=0.05). It was found to be significantly associated with retinoblastomas showing tumor cells invading the optic nerve and choroid than those remaining in situ (p=0.0005). Cellular study further demonstrated that BMI-1 regulated retinoblastoma Y79 cell proliferation and the formation of multicellular spheres, the cell cycle, and apoptosis. This was mediated by the regulation of PCNA, cyclin D1 and D2, and CDK inhibitors p14ARF and p16INK4. Hence, our findings indicate that BMI-1 might play an important role in the development of and malignancy of human retinoblastoma.

Retinoblastoma results from *RB1* gene inactivation and loss of pRB1, yet the proliferation and metastasis that underlie retinoblastoma development have not been well defined. In this study, we showed that BMI-1, a polycomb group transcription factor and an oncogene, plays a crucial role in retinoblastoma proliferation, and may contribute to its malignancy. Its expression was significantly increased when tumor cells were found in the optic nerve and/or choroid (pT3 grade) than in those confined to the retina (pT1 grade). This pathological grading follows the TNM Classification for Retinoblastoma. This suggests a role of BMI-1 in retinoblastoma progression; further, it has the potential to be developed as a novel diagnostic marker of retinoblastoma. This observation is consistently found in other cancers, such as metastatic melanoma and cancers of the pancreas, cervix, breast, and liver [29,31,32,37-39].

In addition to a role in tumorigenesis, BMI-1 maintains cancer (stem) cell identity in leukemia, multiple myeloma, breast, lung, and prostate cancer [23,24]. The importance of these stem-like cells has been proposed for retinoblastoma growth [14,40]. Small populations of retinoblastoma cells have been found to express stem cell–associated markers SOX2, aldehyde dehydrogenase 1, p63, and ABCG2, and to exclude Hoechst dye [15,16]. It is probable that retinoblastoma glial cells, which constitute 2 to 3% of cells in tumors, are the cancer stem cell population and the origin of tumor cell proliferation [41]. These cells express N-MYC and MDM2, which suppresses ARF-induced apoptosis and promotes cell proliferation cone-specific retinoid X receptor alpha (RXRα) signaling [42]. Our finding of elevated BMI-1 expression, particularly in undifferentiated retinoblastomas, further indicates the self-renewal capacity and extensive proliferation of these tumor cells. The detection of retinal development–related genes, including *Nestin*, *PAX6*, *Rx*, and *CHX10* reveals their multipotency [16]. In our study, the upregulation of *CHX10* and *Rx* in the event of BMI-1 overexpression in Y79 cells also indicates BMI-1 involvement in retinal development.

In Y79 cells, BMI-1 upregulated cell proliferation markers cyclin D1, D2, and PCNA, and inhibited of p14ARF and p16INK4. We suggest that Y79 proliferation could be controlled by BMI-1 via its regulation of canonical cyclin targets and CDK inhibitors. Although there was no significant change in cells recruited to the S-phase, the expression of BMI-1 substantially reduced Y79 cell apoptosis, probably mediated by its abrogation of p14ARF and p16INK4. All such effects were reversed by specific BMI-1 knockdown. In common with other cancer cells, our findings reveal that BMI-1 is important for retinoblastoma cell growth.

In conclusion, we identified significant BMI-1 upregulation in retinoblastomas of low differentiation status and invasion to posterior tissues. BMI-1 might be developed as a potential diagnostic marker and an important therapeutic target for retinoblastoma through specific inhibition of BMI-1 by small interfering RNAs.
